# Dual Role of Natural Killer Cells on Graft Rejection and Control of Cytomegalovirus Infection in Renal Transplantation

**DOI:** 10.3389/fimmu.2017.00166

**Published:** 2017-02-16

**Authors:** Miguel López-Botet, Carlos Vilches, Dolores Redondo-Pachón, Aura Muntasell, Aldi Pupuleku, José Yélamos, Julio Pascual, Marta Crespo

**Affiliations:** ^1^Hospital del Mar Medical Research Institute (IMIM), Barcelona, Spain; ^2^Department of Immunology, Hospital del Mar, Barcelona, Spain; ^3^Univ. Pompeu Fabra, Barcelona, Spain; ^4^Immunogenetics-Histocompatibility, Instituto de Investigación Sanitaria Puerta de Hierro, Majadahonda, Spain; ^5^Department of Nephrology, Hospital del Mar, Barcelona, Spain

**Keywords:** human, natural killer, cytomegalovirus, renal, transplantation, rejection

## Abstract

Allograft rejection constitutes a major complication of solid organ transplantation requiring prophylactic/therapeutic immunosuppression, which increases susceptibility of patients to infections and cancer. Beyond the pivotal role of alloantigen-specific T cells and antibodies in the pathogenesis of rejection, natural killer (NK) cells may display alloreactive potential in case of mismatch between recipient inhibitory killer-cell immunoglobulin-like receptors (KIRs) and graft HLA class I molecules. Several studies have addressed the impact of this variable in kidney transplant with conflicting conclusions; yet, increasing evidence supports that alloantibody-mediated NK cell activation *via* FcγRIIIA (CD16) contributes to rejection. On the other hand, human cytomegalovirus (HCMV) infection constitutes a risk factor directly associated with the rate of graft loss and reduced host survival. The levels of HCMV-specific CD8^+^ T cells have been reported to predict the risk of posttransplant infection, and KIR-B haplotypes containing activating KIR genes have been related with protection. HCMV infection promotes to a variable extent an adaptive differentiation and expansion of a subset of mature NK cells, which display the CD94/NKG2C-activating receptor. Evidence supporting that adaptive NKG2C^+^ NK cells may contribute to control the viral infection in kidney transplant recipients has been recently obtained. The dual role of NK cells in the interrelation of HCMV infection with rejection deserves attention. Further phenotypic, functional, and genetic analyses of NK cells may provide additional insights on the pathogenesis of solid organ transplant complications, leading to the development of biomarkers with potential clinical value.

## Introduction

Kidney transplantation is a widely used therapeutic intervention for chronic renal failure. Graft rejection remains a major complication, requiring prophylactic/therapeutic administration of immunosuppressive drugs. Consequently, kidney transplant recipients (KTR) are exposed to an increased susceptibility to infections, particularly by herpesviruses (e.g., cytomegalovirus and Epstein–Barr virus). Besides the pivotal role played by alloantigen-specific T cells and antibodies in the pathogenesis of graft rejection, natural killer (NK) cells alloreactivity and their contribution to antiviral defense receive increasing attention.

### Diversity of the Human NK Cell Receptor Repertoire and NK Cell Subsets Distribution

Natural killer cells constitute an innate lymphoid lineage involved in early defense against certain intracellular pathogens and tumors, which mediate cytotoxicity and pro-inflammatory cytokine production upon interaction with pathological cells (1–3). NK cells are controlled by an array of germ line-encoded inhibitory and activating/co-stimulatory receptors (NKR), as well as by different cytokines (e.g., IL-2, IL-12, IL-15, IL-18, and type I interferons), which regulate their differentiation, proliferation, and effector functions. Inhibitory killer-cell immunoglobulin-like receptors (KIRs) and CD94/NKG2A complement each other, scanning potential target cells for altered surface expression of HLA class I (HLA-I) molecules.

The combinatorial distribution of these NKR along differentiation determines the existence of a variety of NK cell subsets capable of responding against pathological cells, which have downregulated HLA-I expression, as predicted by the “missing-self” hypothesis (4). In the context of transplantation, NK cell subsets may also react against normal allogeneic cells lacking specific HLA-I ligands for their inhibitory KIR (iKIR).

Killer-cell immunoglobulin-like receptor and NKG2 NK cell receptor families include other members with activating function whose physiological role is being investigated. At late differentiation stages, cytolytic T lymphocytes (TCRαβ CD8^+^, CD4^+^, and TCRγδ) may also display HLA-specific NKR (i.e., KIR, CD94/NKG2A, CD94/NKG2C, and LILRB1) (5, 6).

### KIRs for HLA-A, -B, and -C

The human KIR family comprises (i) six receptors (four KIR2DL and two KIR3DL) with cytoplasmic “immunoreceptor tyrosine-based inhibition motifs” (ITIMs), which recruit the SHP-1/2 tyrosine phosphatases preventing NK cell activation; (ii) six KIR with short cytoplasmic tails lacking ITIMs (i.e., KIR2DS and KIR3DS), which interact with DAP12; this adaptor molecule contains “immunoreceptor tyrosine-based activation motifs” (ITAM) linked to protein tyrosine kinase (PTK) activation pathways; and (iii) two KIR (2DL4 and 3DL3) displaying ambiguous signaling motifs (7, 8).

Most iKIRs specifically recognize sets of HLA class Ia (i.e., HLA-A, -B, and -C) allotypes sharing structural polymorphisms at the α1 domain; yet, the ligands for some of them (e.g., KIR2DL5) and most activating KIR (aKIR) remain elusive. In an example of convergent evolution, the physiological role of KIR is undertaken in mice by members of the Ly49 lectin-like family; the Ly49H receptor triggers NK cell functions upon interaction with the m157 viral protein, contributing to defense against murine CMV (9–11). The low affinity interaction of some aKIR with HLA-I molecules suggests that they might specifically recognize pathogen-derived HLA–peptide complexes or other as yet unknown molecules.

At the population level, KIR repertoires are quite diverse due to the fact that not all KIR loci are found in the genome of every individual, and to the existence of a variety of alleles. Each KIR is encoded by a different gene in chromosome 19q13.4, and multiple KIR haplotypes/genotypes have been described worldwide (8). Moreover, iKIR–ligand interactions modulate functional NK cell maturation through an education process termed “licensing,” ill-defined at the molecular level, which dictates that most mature NK cells display at least an inhibitory NKR specific for self HLA-I molecules (12, 13).

### CD94/NKG2 Killer Lectin-Like Receptors for HLA-E

CD94 and members of the NKG2 family are lectin-like membrane glycoproteins encoded at the NK gene complex on human chromosome 12. Similar to KIRs, the CD94/NKG2A heterodimer constitutes an inhibitory receptor linked to the SHP-1 tyrosine phosphatase, and CD94/NKG2C is coupled through DAP12 to a PTK activation pathway (14). The specific ligand for both CD94/NKG2 receptors is constituted by the HLA-E class Ib molecule, which binds to leader sequence peptides from other HLA-I molecules, including alleles not recognized by iKIRs (15–17). Thus, CD94/NKG2A prevents the response against cells with a normal expression of HLA-I molecules, complementing the function of KIRs. HLA-E may present pathogen-derived peptides [e.g., human cytomegalovirus (HCMV), HIV-1, and HCV] altering CD94/NKG2A recognition (18–20). On the other hand, CD94/NKG2C binds to HLA-E with lower affinity than its inhibitory counterpart (21, 22) and has been reported to be involved in the response to human HCMV (see Adaptive NK Cell Response to HCMV).

### Additional Activating and Inhibitory NKR

The CD16A (FcγRIIIA) receptor is coupled through CD3ζ or FcεRIγ chain adapters to a PTK activation pathway, triggering cytotoxicity and cytokine production upon interaction with IgG-opsonized cells (23). A CD16A allelic dimorphism (158V or F) influences the affinity of its interaction with IgG, modulating receptor-mediated signaling and activation of effector functions (24). Surface CD16 expression is downregulated in activated NK cells through a shedding process mediated by ADAM-17 metalloprotease (25, 26).

The human NKG2D C-type lectin triggers phosphatidyl inositol-3 kinase signaling through the DAP10 adaptor (27). NKG2D functions as an activating/co-stimulatory receptor specific for a set of ligands (MICA, MICB, and “UL16-binding proteins”) displayed by pathological cells, which are also inducible by cellular stress in normal tissues (6). Several immune evasion mechanisms that prevent NKG2D ligand (NKG2D-L) expression in HCMV-infected cells have been identified (28).

Natural cytotoxicity receptors, i.e., NCR1 (NKp46), NCR2 (NKp44), and NCR3 (NKp30), are connected to PTK signaling pathways through different ITAM-bearing adapters (29). In addition to their putative role in recognition of pathogen-derived molecules, there is evidence supporting the expression of ligands in normal cells that may trigger NK cell functions when control by inhibitory receptors is reduced (30). NKp46 is coupled to the CD3ζ or FcεRIγ chain, triggering cytotoxicity and cytokine production upon recognition of an ill-defined cellular ligand(s). NKp46 has been shown to be involved in the NK cell response to HCMV-infected dendritic cells and macrophages (31, 32). The nature of cellular ligands for NKp44 also remains open, and several ligands have been reported for the CD3ζ-linked NKp30 (30, 33).

In addition to the pivotal role played by adhesion molecules (i.e., LFA-1 and CD2) in the NK cell interaction with target cells, engagement of DNAM1, a co-stimulatory receptor specific for Nectin-2 (CD112) and PVR (CD155), contributes to the response against tumor and virus-infected cells (32, 34). NK cells may acquire additional inhibitory NKR upon activation or at late differentiation stages. Among these checkpoints, LILRB1 (ILT2, LIR-1, or CD85j) interacts with a wide spectrum of HLA-I molecules and binds with a higher affinity to the UL18 HCMV glycoprotein (35, 36); similarly, TIGIT (T cell Ig and ITIM domain) binds to CD155 competing with DNAM1 (37).

### Peripheral Blood NK Cell Subsets

**Figure 1:**
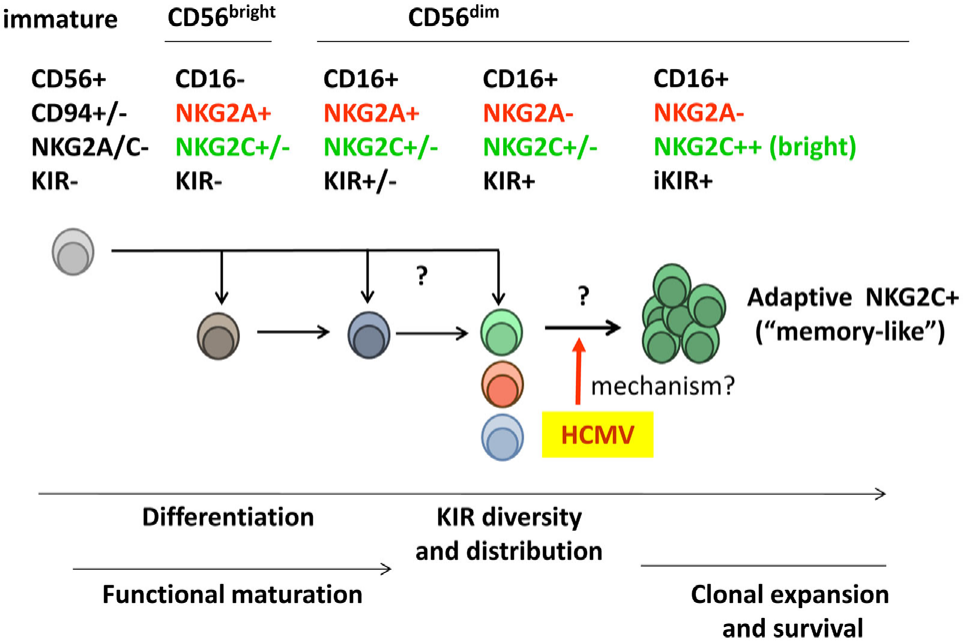
**Diversity of the human peripheral blood natural killer (NK) cell compartment**. Two main NK cell populations are identified according to expression levels of the CD56 marker. CD56^bright^ NK cells secrete pro-inflammatory cytokines but display a low cytotoxic potential and are often considered to represent an early maturation stage. According to such view, this subset is believed to differentiate into the major cytotoxic CD56^dim^ NK cell population, which includes a variety of subsets differing in NKR expression [e.g., killer-cell immunoglobulin-like receptors (KIRs), CD94/NKG2A, and CD94/NKG2C]. Whether some CD56^dim^ subsets (e.g., NKG2A^−^ KIR^+^) might directly derive from immature NK cell precursors rather than from NKG2A^+^ KIR^−^ CD56^bright^ NK cells is not formally ruled out. Human cytomegalovirus (HCMV) infection promotes the differentiation and stable expansion of an NK cell subset, which displays high levels of the CD94/NKG2C receptor and an oligoclonal inhibitory KIR (iKIR) expression pattern, associated with other phenotypic and functional characteristics (see details in Section “Adaptive NK Cell Response to HCMV”). The nature of their precursors and the mechanism(s) underlying such adaptive NK cell response to HCMV are investigated.

The human peripheral blood NK cell compartment includes a variety of cell subsets, which represent distinct maturation stages and display different combinations of HLA-I-specific NKR. Similar to T and B lymphocytes, NK cells may undergo clonal expansion and late differentiation events, skewing the NKR repertoire and further diversifying their phenotypic/functional profile (Figure 1).

Two NK cell populations are identified in peripheral blood according to their surface expression levels of the CD56 neural-cell adhesion molecule isoform (i.e., CD56^bright^ and CD56^dim^) (38). CD56^bright^ NK cells constitute a minor fraction (~10%) of the normal circulating NK cell compartment. They display a low cytotoxic potential but secrete pro-inflammatory cytokines and are conventionally considered to represent an early maturation stage (39). Most CD56^bright^ NK cells express CD94/NKG2A, NKG2D, and NCR, but lack KIR and CD16. The predominant (~90%) CD16^+^NKG2D^+^CD56^dim^ NK cell population comprises distinct subsets, defined according to KIR, NKG2A, and NKG2C expression (e.g., NKG2A^+^KIR^+^NKG2C^+/−^ and NKG2A^−^KIR^+^NKG2C^+/−^). Evidences have been obtained indirectly supporting a linear differentiation model in which CD56^bright^ NK cells sequentially give rise to the other NK cell subsets (38, 40). Yet, the possibility that alternative differentiation pathways branching from NK cell precursors may independently generate CD56^bright^ and CD56^dim^ subsets cannot be formally ruled out.

Further levels of NK cell phenotypic/functional heterogeneity are determined by (i) the diversity of human NKR repertoires, conditioned by the existence of hundreds of different KIR haplotypes diverging in gene and allotype content; (ii) the clonal distribution of KIR combinations among CD56^dim^ NK cells, modulated by the influence of KIR–ligand interactions on NK cell maturation; (iii) the oligoclonal adaptive expansion of NK cell subsets in response to HCMV infection (see Adaptive NK Cell Response to HCMV); and (iv) the incidence of late differentiation events, which determine additional phenotypic and functional changes (e.g., expression of CD57 and LILRB1) (Figure 1).

## NK Cells and HCMV Infection in KTR

Human cytomegalovirus is a member of the herpesviridae family which causes highly prevalent lifelong infections in all human populations, generally asymptomatic in immunocompetent hosts. The virus establishes latency, undergoing occasional reactivation which allows its efficient transmission through secretions (41, 42). HCMV may cause severe congenital disorders (43) and increases the morbidity/mortality rate in immunocompromised individuals (44, 45), being associated with some chronic inflammatory disorders (i.e., atherosclerosis) and immune senescence (46). As a consequence of immunosuppression to prevent graft rejection, KTR are exposed to HCMV reactivation/reinfection, leading to potentially severe complications (47, 48).

Together with specific T lymphocytes and antibodies, commonly analyzed to assess the adaptive immune response to HCMV, NK cells contribute to defense against this pathogen (49, 50). To escape from CD8^+^ T cells, HCMV downregulates surface expression of HLA-I molecules in infected cells, interfering with antigen presentation (51, 52). Consequently, engagement of inhibitory NKR is impaired promoting NK cell activation, which is counteracted by a variety of viral immune evasion strategies (53–55).

### Adaptive NK Cell Response to HCMV

In 2004, we discovered that healthy HCMV-seropositive (HCMV^+^) individuals display increased proportions of NK and T cells hallmarked by high surface levels of CD94/NKG2C (NKG2C^bright^) (56). The imprint of HCMV in the NK cell compartment is perceived to a variable extent only in some HCMV^+^ subjects, persisting under steady state conditions. A number of reports have extended these observations in different settings, and the terms “adaptive” or “memory-like” are currently employed to designate the human differentiated NKG2C^bright^ NK cell population (55). For the sake of precision, we have strictly used this original definition along the text. Yet, it is of note that these terms have been used by some authors to define other NK cell populations (e.g., *in vitro* cytokine-differentiated NK cells) (57).

Expansions of NKG2C^bright^ cells are not induced by other herpesviruses (i.e., EBV and HSV-1) but have been reported in the course of different viral infections, yet associated with HCMV coinfection (58–61). As compared to other NK cell subsets, including the low proportions of NKG2C^dim^ cells detected in HCMV(−) and some HCMV(+) individuals, adaptive NKG2C^+^ NK cells display a phenotype characterized by an oligoclonal pattern of iKIR specific for self HLA-I molecules (preferentially HLA-C). Moreover, they express reduced levels of NCR (i.e., NKp30 and NKp46), Siglec7, and CD161 (56, 62–64), acquire late differentiation markers (e.g., CD57 and LILRB1) (65, 66), maintain surface expression of NKG2D and CD16, and display increased levels of CD2 involved in their activation (67, 68). Epigenetic downregulation of signaling molecules (e.g., FcεRIγ chain and Syk) and certain transcription factors have been associated with adaptive NK cell differentiation (69, 70). From a functional standpoint, they contain greater levels of Granzyme B and efficiently secrete TNF-α and IFN-γ (62, 63), mediating antibody-dependent cytotoxicity (ADCC) and cytokine production against HCMV-infected cells (71–73).

Expansions of NKG2C^+^ cells following HCMV infection were reported in immunosuppressed transplant recipients (65, 66, 74), in a severe T cell primary immunodeficiency (75), as well as in children and newborns with congenital or postnatal HCMV infection (76, 77), independently of aging (78–80). Altogether, these observations suggest that the magnitude of the HCMV imprint on the NK cell compartment in healthy individuals is likely fixed at the time of primary infection, presumably depending on host/virus genetics and other circumstantial factors (e.g., age at infection, viral load, etc.) (81).

**Figure 2:**
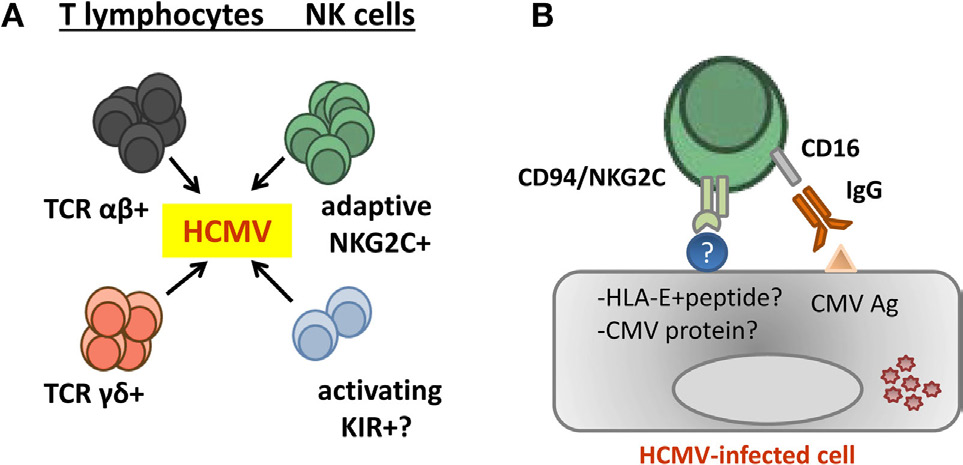
**Contribution of adaptive natural killer (NK) cells to human cytomegalovirus (HCMV) control**. **(A)** Evidences supporting a contribution of different T and NK cell subsets in the control of HCMV infection in kidney transplant recipients have been reported. **(B)** Adaptive NKG2C^bright^ NK cells generated in response to HCMV infection efficiently mediate antibody-dependent cytotoxicity and cytokine production (e.g., TNF-α and IFN-γ) in response to HCMV-infected cells. Yet, there is no consistent evidence supporting an involvement of CD94/NKG2C in triggering NK cell effector functions against infected cells, and the nature of a hypothetical viral ligand remains elusive.

By analogy with the role of Ly49H^+^ cells in the response to murine CMV (82), we hypothesized that CD94/NKG2C-mediated specific recognition of virus-infected cells drives the adaptive differentiation, proliferation, and survival of this lymphocyte subset (55). Indirectly supporting this view, *in vitro* stimulation of PBMC from HCMV^+^ donors with virus-infected cells elicited a preferential expansion of CD94/NKG2C^+^ NK cells (83, 84). Yet, at variance with Ly49H, the nature of a hypothetical viral ligand remains uncertain, and there is no experimental evidence supporting that the CD94/NKG2C receptor may trigger NK cell effector functions against HCMV-infected cells (32, 55, 83, 85). By contrast, NKG2C^+^ adaptive NK cells have been shown to efficiently mediate antibody-dependent effector functions, particularly pro-inflammatory cytokine production, against HCMV and HSV-1 infected cells (24, 71). It is of note that CD16 remains functionally coupled to the CD3ζ adapter (73) following downregulation of FcεRIγ. The molecular mechanisms driving this pattern of response to HCMV and the existence of a putative CD94/NKG2C viral ligand are investigated (Figure 2).

A deletion of the *NKG2C* gene (officially designated *KLRC2*) is frequently detected in different human populations, with some variation depending on their ethnic/geographic origin (86–89). *NKG2C* gene copy number is directly related with surface expression levels and the activating function of CD94/NKG2C (62). Moreover, the *NKG2C* genotype is as well associated with steady state numbers of circulating NKG2C^+^ NK cells, which appear reduced in *NKG2C*^+/del^ as compared to *NKG2C*^+/+^ individuals, further supporting a role of the NKR in driving the generation of adaptive NK cells (62, 76, 88). The identification of ~5% HCMV(+) healthy *NKG2C*^del/del^ blood donors illustrates that the receptor is dispensable for controlling the viral infection under normal conditions, being redundant with other cell types (i.e., T lymphocytes). Moreover, NKG2C^−^ NK cell subsets sharing some phenotypic features with canonical adaptive NKG2C^+^ NK cells have been reported in HCMV(+) *NKG2C*^del/del^ blood donors (68, 90) and HCMV-infected hematopoietic stem cell transplantation (HSCT) recipients (91). On the other hand, the lack of NKG2C^+^ NK cells has been suggested to alter the control of primary HCMV infection in childhood (88); a putative relevance of the *NKG2C* deletion in immunosuppressed patients is discussed in the next section.

### NK Cell Response to HCMV Infection in KTR

Posttransplant HCMV infection constitutes a risk factor for cardiac and renal allograft vasculopathy associated with chronic graft dysfunction and is directly associated with the rate of graft loss and reduced host survival (47, 48, 92). Antiviral prophylaxis is commonly administered to HCMV(−) KTR transplanted from an HCMV(+) donor or treated with intensive immunosuppression; patients developing HCMV viremia receive antiviral therapy, not free of adverse effects. Identification of biomarkers predicting the risk of posttransplant HCMV infection is warranted to improve its clinical management. Regular immunosuppressive therapy in KTR is aimed to prevent rejection, impairing the development of alloreactive T cells and production of alloantibodies, but has been proposed to be less effective on differentiated CTL and mature NK cells (93). Yet, alterations of the phenotypic and functional profile of circulating NK cells following immunosuppression were detected in other studies (94, 95). After low-dose therapy with anti-thymocyte globulin (ATG) NK cells recovered faster than T cells (96). In this regard, following induction with ATG functionally competent NK cells were reported to display for several months an NKG2A^+^ KIR^−^ phenotype (97). Thus, it is plausible that NK cells may contribute to antiviral defense in KTR, partially compensating their impaired T cell response.

The putative influence of KIR and HLA-I genotypes in the control of HCMV infection in KTR has been addressed. A relation of the KIR repertoire with viral load was reported in primary HCMV infection (98), even though the risk of HCMV disease was not influenced by KIR–ligand matching (99). De Rham et al. detected increased numbers of KIR3DL1^+^ NK cells in KTR during the acute phase of HCMV reactivation (100). In both KTR and healthy blood donors, this NK cell subset efficiently killed *in vitro* infected fibroblasts; different interpretations for this observation were proposed. On the other hand, KIR-B haplotypes encoding aKIR were related with a lower rate of HCMV infection (101). In cases receiving thymoglobulin and intensive immunosuppression, KIR-associated control of HCMV was limited to seropositive KTR (102). A role of activating NKR in the control of other viral infections (e.g., BK and varicella zoster) has been also proposed (103, 104).

We recently explored the relationship of adaptive NKG2C^+^ NK cells with the outcome of HCMV infection in KTR, monitoring pre- and posttransplant the NK cell immunophenotype and the incidence of viremia (105). NKG2C^+^ NK cell expansions did not systematically follow detection of HCMV viremia in KTR, thus suggesting that a prompt control of the infection by antiviral therapy and preexisting differentiated CTL may hamper the adaptive NK cell response development. Conversely, late NKG2C^+^ NK cell expansions might reflect clinically unnoticed HCMV replication after withdrawal of antiviral therapy. In this regard, symptom-free HCMV reactivations in KTR have been associated with altered phenotypic and functional profiles of NK cells, which expressed LILRB1 and downregulated FcϵRIγ (106). In the same line, increased proportions of LILRB1^+^ (LIR-1^+^) NK cells were originally associated with HCMV infection in lung transplant recipients (107).

Regular immunosuppressive protocols did not modify the levels of adaptive NK cells in KTR without detectable viremia along the follow-up, nor did they impair their expansion in some cases undergoing HCMV infection (105). Nevertheless, the possibility that immunosuppression may interfere with *de novo* adaptive NK cell differentiation, as it does with alloreactive T cell development, is not ruled out. Further studies are warranted to precisely assess the impact of different drugs on the development and effector functions of adaptive NK cells.

Of note, high pretransplant levels of NKG2C^+^ NK cells were associated with a reduced incidence of posttransplant HCMV viremia, independently of other related variables (e.g., thymoglobulin induction, antiviral prophylaxis, and age), suggesting that adaptive NK cells might confer some protection against viral reactivation/reinfection (105). In this regard, a low NK cell count post-liver transplantation has been reported to be an independent risk factor for HCMV disease (108). Despite their limited direct *in vitro* response against HCMV-infected cells, adaptive NKG2C^+^ NK cells may contribute to antiviral defense. In particular, they efficiently mediate antibody-dependent effector functions and likely participate in the response to HCMV reactivation in KTR, in combination with specific IgG (70, 71, 73) (Figure 2B). In this context, the influence of CD16A dimorphism and IgG allotypes on the magnitude of ADCC deserves attention (24). The possibility that aKIR may be involved in the putative antiviral effect of adaptive NKG2C^+^ NK cells appears unlikely, considering that they do express iKIR (63, 64, 90) and that their expansion is independent of KIR-A/B haplotypes (56). Nevertheless, NK cell subsets expressing CD94/NKG2C or aKIR might play complementary roles in the response to HCMV.

**Figure 3:**
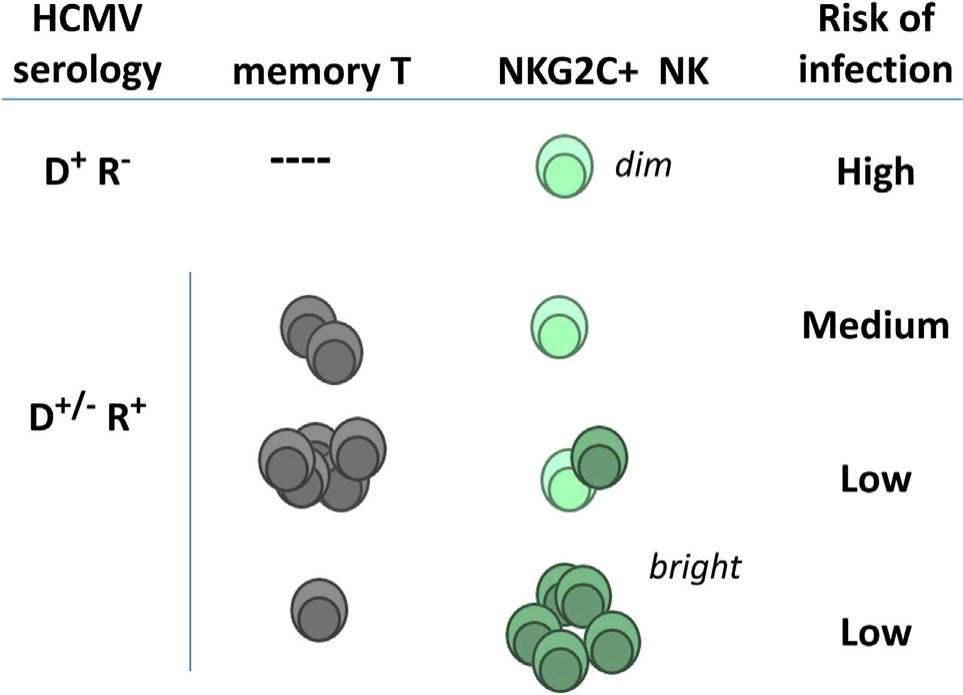
**Hypothetical relation of adaptive natural killer (NK) cells and specific T lymphocytes with posttransplant infection risk in kidney transplant recipients**. NKG2C^dim^ NK cells detected in human cytomegalovirus (HCMV)(−) individuals express lower surface levels of the receptor and differ phenotypically and functionally from adaptive NK cells expanding in response to HCMV infection (NKG2C^bright^) (see details in Section “Adaptive NK Cell Response to HCMV”). Pretransplant frequencies of virus-specific T lymphocytes and of NKG2C^+^ NK cells in seropositive recipients have been independently related with a reduced incidence of HCMV infection (D, donor; R, recipient).

The frequencies of TcRαβ T cells specific for HCMV antigens (e.g., IE-1 and pp65) have been reported to predict the risk of posttransplant infection (109, 110); moreover, TcRγδ T cells were associated with control of posttransplant HCMV viremia (111). Adaptive NKG2C^+^ NK cells and CTL have been proposed to be independent (78–80). Thus the possibility that the association of adaptive NKG2C^+^ NK cells with a lower risk of HCMV infection might indirectly reflect a central role of HCMV-specific TcRαβ T cells (Figure 3) appears unlikely; further studies are warranted to precisely address this issue.

The distributions of the *NKG2C* genotypes in two different KTR cohorts, studied pre- and posttransplant, appeared comparable to the frequencies detected in blood donors; as reported, the magnitude of the NKG2C^+^ NK cell expansion was greater in *NKG2C*^+/+^ than in *NKG2C*^+/del^ subjects (105). Remarkably, somewhat increased frequencies of the *NKG2C*^+/del^ genotype and a reciprocal reduction of *NKG2C*^+/+^ cases were detected among KTR suffering symptomatic HCMV infection; unexpectedly, an opposite reduction of the NKG2C^del/del^ frequency was observed among this KTR group. Despite that differences did not reach statistical significance, the coincident trends in both cohorts suggested a relation of *NKG2C* copy number with the outcome of HCMV infection and its impact in KTR; larger studies are warranted to confirm these observations.

Altogether these results indirectly support that adaptive NKG2C^+^ NK cells may play an active role in defense against HCMV, partially compensating in KTR the effect of immunosuppression on T cells. High pretransplant levels of NKG2C^+^ cells may predict a lower risk of posttransplant HCMV replication/disease in KTR receiving regular immunosuppression, particularly in *NKG2C*^+/+^ HCMV(+) patients (Figure 3). On the other hand, posttransplant expansions of differentiated adaptive NKG2C^+^ NK cells reflect the incidence of viral replication and, once established, might contribute to its control. It is plausible that antibody-mediated response to other viral infections may as well contribute to the expansion of adaptive NKG2C^+^ cells (70, 112). It is uncertain whether adaptive NK cells may comparably respond to HCMV reactivation or reinfection, reported to have a different clinical impact (113). From a practical standpoint, monitoring basal and posttransplant levels of adaptive NK cells may provide biomarkers to evaluate the control of HCMV, with practical implications in the clinical management of the viral infection. Assessing the relation with other phenotypic features displayed at late stages of adaptive NKG2C^+^ NK cells differentiation (e.g., CD57 expression and FcεRIγ chain loss) deserves attention. Furthermore, studies in larger cohorts are required to assess the relation of the adaptive NK cell response in KTR with the incidence of other viral infections, as well as with the risk of chronic graft rejection, cardiovascular disease, and cancer (48, 114). In this regard, the possibility that antibody-dependent activation of adaptive NK cells may participate in donor-specific alloantibodies (DSA)-mediated rejection is addressed in the next section.

## NK Cells and Allograft Rejection

### NK Cell Alloreactivity

**Figure 4:**
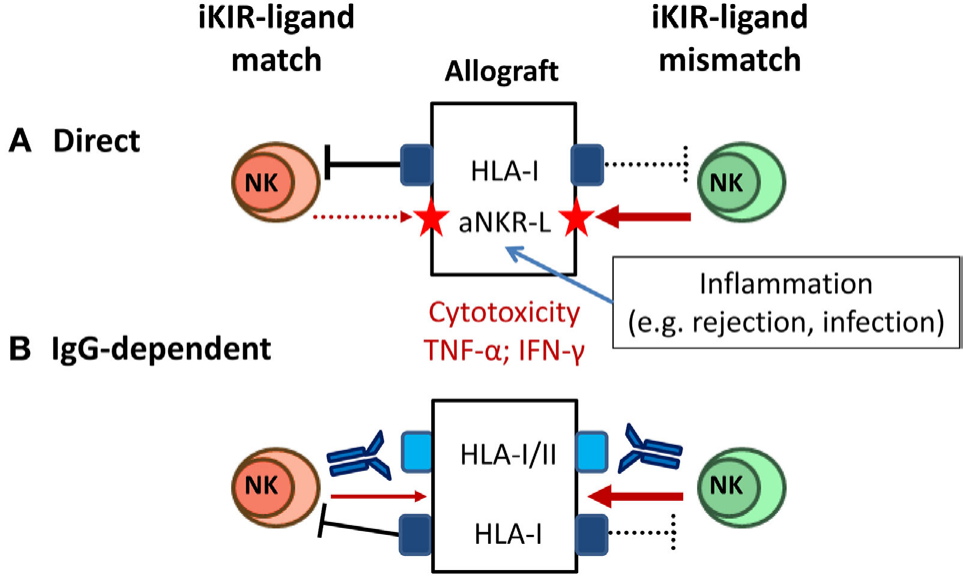
**Natural killer (NK) cell-mediated alloreactivity in solid organ transplantation**. **(A)** NK cells lacking inhibitory KIR (iKIR) specific for donor HLA class I ligands (“mismatched,” right) may potentially mediate cytotoxicity and cytokine production against the allograft. NK cell alloreactivity is favored by cellular stress conditions (e.g., pro-inflammatory stimuli) inducing graft expression of ligands for activating NKR (e.g., NKG2D ligand). **(B)** Donor-specific alloantibodies (DSA) trigger CD16^+^ NK cell-mediated cytotoxicity and cytokine production against the allograft, overcoming the control by inhibitory NKR (e.g., iKIR) (left); iKIR–ligand mismatch might synergize with a DSA-CD16-mediated response (right).

Mature NK cells whose iKIR fail to recognize HLA-I alleles on an allograft are predicted to mediate cytotoxicity and pro-inflammatory cytokine production, as long as activating NKR are engaged by ligands displayed on target cells. Some degree of KIR–ligand mismatching between donors and recipients is estimated to occur in 50–75% of HLA non-identical transplants, and several studies have addressed the impact of this variable in kidney transplant outcome (Figure 4A). On one hand, KIR–ligand mismatches were suggested to influence short-term outcome in KTR (115) and were associated with a reduced long-term graft survival in HLA-incompatible KTR (116), proposing a beneficial effect of NK cell-targeted immunosuppression. Conversely, KIR–ligand mismatch was reported by others to be irrelevant for predicting long-term allograft survival (117) and, in the same line, no effect on the risk of rejection was perceived after reduction of immunosuppressive therapy (118).

These apparently conflicting observations might be reconciled considering the implications of inhibitory NKR–MHC class I mismatch in other experimental and clinical transplant settings. Classical animal models of “F1-hybrid resistance” revealed a role of NK cells in rejection of allogeneic hematopoietic transplants, but not of other tissues grafts (119). In HSCT, donor NK cell-mediated alloreactivity has been shown to potentially exert an antileukemic effect without promoting graft-versus-host disease (120). In the same line, adoptive immunotherapy with allogeneic NK cells in HSCT recipients has been proven a safe procedure (121, 122). Altogether these observations support that the NK cell alloreactive potential, determined by KIR–ligand mismatch, may have a negligible pathogenic impact in solid organ transplantation, unless engagement of activating NKR triggers NK cell effector functions. Accordingly, NK cell alloreactivity would be favored by stimuli promoting graft expression of activating NKR ligands (e.g., NKG2D-L). This situation may take place in the context of infections (e.g., HCMV) or T cell/DSA-mediated rejection reactions, enhancing the pathogenic impact of these adverse events (Figure 4A). From a methodological standpoint, the genotypic prediction of KIR–ligand mismatching should be complemented by a direct assessment of the frequencies of potential alloreactive NK cells, using specific mAbs to discriminate homologous activating and iKIR as reported for HSCT (123).

### Alloantibody-Dependent NK Cell Activation

Posttransplant donor-specific anti-HLA antibodies (DSA) are a major risk factor in kidney transplant, causing microvascular damage associated with humoral rejection. In addition to complement activation, HLA-specific alloantibodies may trigger NK cells through CD16 to mediate ADCC and cytokine production (Figure 4B). Indications that NK cells contribute to chronic antibody-mediated rejection (ABMR) have been obtained in experimental models and analyzing kidney biopsies (124, 125). Consistent with a pathogenic role of NK cells, increased CD56^+^ cells have been observed in graft lesions from patients suffering ABMR. NK cell-associated gene expression has been associated with microvascular inflammation (126, 127), providing biomarkers with potential diagnostic/prognostic value (128–130). CD16A is also expressed by TCRγδ and some TCRαβ T lymphocyte subsets (131, 132). CD16^+^ TCRγδ T cells have been related with the response to posttransplant HCMV infection in KTR, and evidences supporting their involvement in ABMR have been reported (111, 133).

CD16 downregulation and expression of activation markers have been observed in circulating NK cells from KTR, likely reflecting IgG-dependent NK cell activation triggered by infectious pathogens (e.g., HCMV) or DSA (134). In the same line, altered distributions of circulating NK cells have been associated with the presence of alloantibodies in KTR. DSA^+^ patients were reported to display lower proportions of the major CD56^dim^ NK cell subset as compared with cases without anti-HLA antibodies (95). Increased proportions of CD56^bright^ and CD56^dim^ NKG2A^+^ cells, but not their absolute numbers, were observed in DSA^+^ KTR (135). The data suggest that alloantibody-mediated activation of NK cells *via* CD16 may promote their turnover, accounting for the imbalanced NK cell subset distribution.

This hypothesis predicts that CD56^bright^ NKG2A^+^ CD16^−^ NK cells should be spared from the effect of alloantibodies, consistent with their increased proportions in DSA^+^ KTR. On the other hand, the association of DSA with increased proportions of CD56^dim^NKG2A^+^ NK cells suggests that engagement of CD94/NKG2A by HLA-E, conserved in all individuals, might also dampen the alloantibody effect on this subpopulation. Conversely, KIR–ligand mismatch would add to alloantibody activation of CD56^dim^NKG2A^−^ KIR^+^ NK cells, synergizing with the pathogenic effects of DSA and accelerating their turnover. Given the oligoclonal expression by adaptive NKG2C^+^ NK cells of self-reactive KIR, preferentially specific for HLA-C molecules (63, 64), and their ability to mediate antibody-dependent effector functions (71, 73), it is likely that they may play a relevant pathogenic role in DSA-mediated graft rejection of KIR–ligand-I mismatched transplants.

In summary, consistent evidence has been obtained supporting a functional duality of NK cells in the context of kidney transplantation, reflected by their positive involvement in the response to HCMV infection as opposed to their participation in graft rejection. Further studies integrating phenotypic, functional, and genetic analysis of NK cells should provide valuable insights on the pathogenesis of solid organ transplant complications, leading to the potential development of clinically useful biomarkers.

## Author Contributions

All authors have actively contributed to build up the conceptual framework developed in this review and revised the draft written by ML-B.

## Conflict of Interest Statement

The authors individually declare that the research was conducted in the absence of any commercial or financial relationship that could be construed as a potential conflict of interest.
